# Metabolism of Stilbenoids by Human Faecal Microbiota

**DOI:** 10.3390/molecules24061155

**Published:** 2019-03-23

**Authors:** Veronika Jarosova, Ondrej Vesely, Petr Marsik, Jose Diogenes Jaimes, Karel Smejkal, Pavel Kloucek, Jaroslav Havlik

**Affiliations:** 1 Department of Food Science, Czech University of Life Sciences Prague, Kamycka 129, 165 00 Prague 6–Suchdol, Czech Republic; jarosovaverca@gmail.com (V.J.); czeveselyo@gmail.com (O.V.); marsik@af.czu.cz (P.M.); jose.d.jaimes@gmail.com (J.D.J.); kloucek@af.czu.cz (P.K.); 2 Department of Microbiology, Nutrition and Dietetics, Czech University of Life Sciences Prague, Kamycka 129, 165 00 Prague 6– Suchdol, Czech Republic; 3 Department of Natural Drugs, Faculty of Pharmacy, University of Veterinary and Pharmaceutical Sciences Brno, Palackeho 1946/1, 612 42 Brno, Czech Republic; karel.mejkal@post.cz

**Keywords:** bacteria colon model, fecal fermentation, metabolites, phenolics, polyphenols, stilbenoids, liquid chromatography high resolution mass spectrometry

## Abstract

Stilbenoids are dietary phenolics with notable biological effects on humans. Epidemiological, clinical, and nutritional studies from recent years have confirmed the significant biological effects of stilbenoids, such as oxidative stress protection and the prevention of degenerative diseases, including cancer, cardiovascular diseases, and neurodegenerative diseases. Stilbenoids are intensively metabolically transformed by colon microbiota, and their corresponding metabolites might show different or stronger biological activity than their parent molecules. The aim of the present study was to determine the metabolism of six stilbenoids (resveratrol, oxyresveratrol, piceatannol, thunalbene, batatasin III, and pinostilbene), mediated by colon microbiota. Stilbenoids were fermented in an in vitro faecal fermentation system using fresh faeces from five different donors as an inoculum. The samples of metabolized stilbenoids were collected at 0, 2, 4, 8, 24, and 48 h. Significant differences in the microbial transformation among stilbene derivatives were observed by liquid chromatography mass spectrometry (LC/MS). Four stilbenoids (resveratrol, oxyresveratrol, piceatannol and thunalbene) were metabolically transformed by double bond reduction, dihydroxylation, and demethylation, while batatasin III and pinostilbene were stable under conditions simulating the colon environment. Strong inter-individual differences in speed, intensity, and pathways of metabolism were observed among the faecal samples obtained from the donors.

## 1. Introduction

Stilbenoids are dietary phenolics that occur in a wide range of edible fruits and seeds, such as grapes (*Vitis vinifera*), peanuts (*Arachis hypogaea*), sorghum (*Sorghum bicolor*), and some tree species (*Pinus* spp. and *Picea* spp.) [1]. Resveratrol (3,4′,5-trihydroxystilbene) is the most studied stilbenoid, and is commonly associated with the French paradox, where resveratrol is thought to lower the incidence of coronary heart disease, despite a high intake of saturated fat in the French population [2]. In mice studies, both in vitro and in vivo, resveratrol has shown strong anti-inflammatory activity by a reduction of tumor necrosis factor alpha (TNF-α) and interleukin 1 beta (IL-1β), an increase of interleukin 10 (IL-10), and a reduced expression of prostaglandin E synthase-1 (PGES-1), cyclooxygenase-2 (COX-2), and inducible nitric oxide synthase (iNOS) [3,4]. Therefore, stilbenoid could act as an anti-inflammatory agent through some of the same mechanisms as nonsteroidal antiphlogistic drugs. Resveratrol has also been reported to affect various factors and metabolic targets associated with oxidative stress [1,5,6]. Among these, resveratrol has a strong affinity to quinone reductase 2, with a dissociation constant as low as 35 nM, making it the strongest known inhibitor so far, which, in turn, may regulate the expression of cellular antioxidant enzymes and cellular resistance to oxidative stress [7]. Resveratrol further interacts with a large number of receptors and enzymes that could plausibly make major contributions to its biological effects. Both in vitro and in vivo, resveratrol treatment upregulates mammalian target of rapamycin (mTOR), sirtuin 1 (SIRT1), and adenosine monophosphate-activated protein kinase (AMPK), which influence the regulation of metabolism in multiple tissues [8,9]. However, the in vivo importance is rather more relevant to insects than to mammals. Structural analogs of resveratrol are present in medicinal plants and show significant bioactivity. For instance, piceatannol (3,3′,4′,5-tetrahydroxystilbene) is found in plants such as grapes (*V. vinifera*), passion fruit (*Passiflora edulis*), Japanese knotweed (*Polygonum cuspidatum*), and Norway spruce (*P. abies*) [10]. Compared to resveratrol, piceatannol shows greater biological activity as an inhibitor of COX-2 and of the constitutive photomorphogenesis 9 signalosome (CSN)-associated kinase [11], possibly due to its better solubility in H_2_O. Moreover, piceatannol inhibits the activation of p40 and p56 protein tyrosine kinases and NF-κB [12]. Another analog of resveratrol, pinostilbene (3,4′-dihydroxy-5-methoxystilbene), found in Siberian pine (*P. sibirica*), showed an inhibition of dopamine-induced cell death through extracellular signal–regulated kinase (ERK 1/2) activation in an in vitro study, was shown to alleviate the loss of motor function seen on aging in vivo [13]. Oxyresveratrol (2′,3,4′,5-tetrahydroxystilbene), found in white mulberry (*Morus alba*), exhibited, among other effects, the inhibition of TNF-α production and stronger antioxidant activity than resveratrol [14]. Many of these molecules have been subjected to clinical trials and are being investigated as clinical drugs [15,16,17].

Phenolics are intensively metabolically transformed in intestinal epithelial cells and transported through the basolateral membrane in the form of conjugates [18]. Some typical intestinal epithelium metabolites include *O*-β-glucuronides, 3-*O*-sulfates, or their methoxy-derivatives. However, a large portion of the phenolic compounds escape intestinal absorption and undergo their microbial metabolic conversion in the colon [19]. Colon catabolism is an important phase of the pharmacokinetics of chemical entities in the human body. Its knowledge is an important prerequisite for bioassay validation or for the development of more active substituents. Colonic catabolites might be more biologically relevant forms of the compounds, and their use in bioassays is a more realistic reflection of the compound’s bioactivity. To date, only resveratrol and its fate in the human colon has been investigated, finding three metabolites: dihydroresveratrol, 3,4′-dihydroxy-trans-stilbene, and 3,4′-dihydroxybibenzyl (lunularin) [20]. The bioactivity of dihydroresveratrol, found by in vivo and in vitro studies, includes antioxidant [21] and anti-inflammatory [22] activity.

Thus, the aim of the present study was to investigate whether six selected stilbenoids (batatasin III, oxyresveratrol, piceatannol, pinostilbene, resveratrol, thunalbene) undergo metabolic transformation by human colon microbiota and thereafter detect their main metabolites by liquid chromatography mass spectrometry (LC/MS).

## 2. Results

**Figure 1:**
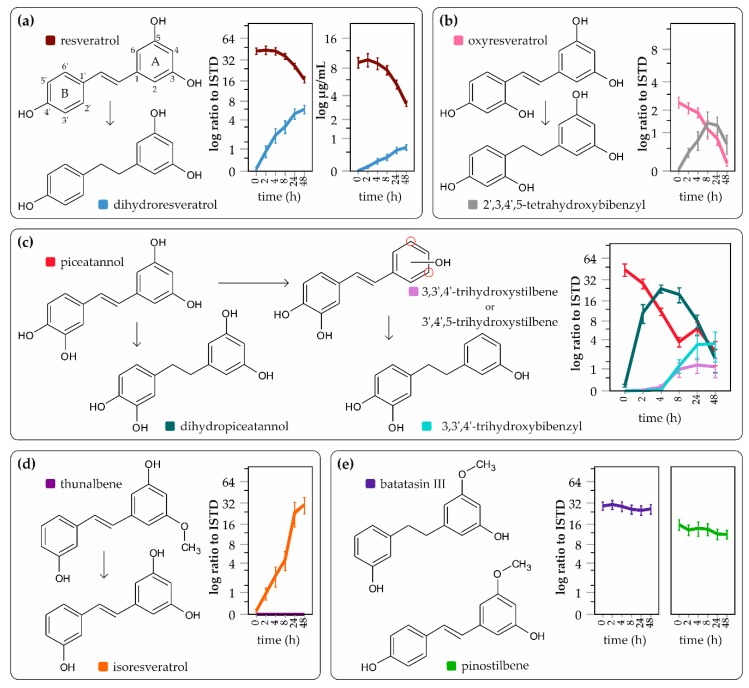
The effect of bacterial metabolism on (**a**) resveratrol (*N* = 5); (**b**) oxyresveratrol (*N* = 3); (**c**) piceatannol (*N* = 5); (**d**) thunalbene (*N* = 5); (**e**) batatasin III (*N* = 4); and pinostilbene (*N* = 5); values obtained from LC/MS are expressed as ratios of produced metabolite to internal standard (ISTD) as means ± 1 SE (*p* < 0.05); N represents the number of donors analyzed.

The in vitro faecal fermentation system, using fresh faeces from five different donors (D) as inoculum, was performed to analyze the metabolism of selected stilbenoids (batatasin III, oxyresveratrol, piceatannol, pinostilbene, resveratrol, thunalbene) in the human colon. As seen in Figure 1, significant differences in the microbial transformation among stilbene derivatives were observed. Four stilbenoids (resveratrol, oxyresveratrol, piceatannol and thunalbene) were metabolically transformed to new products, while batatasin III and pinostilbene were stable in the colon environment.

**Figure A1:**
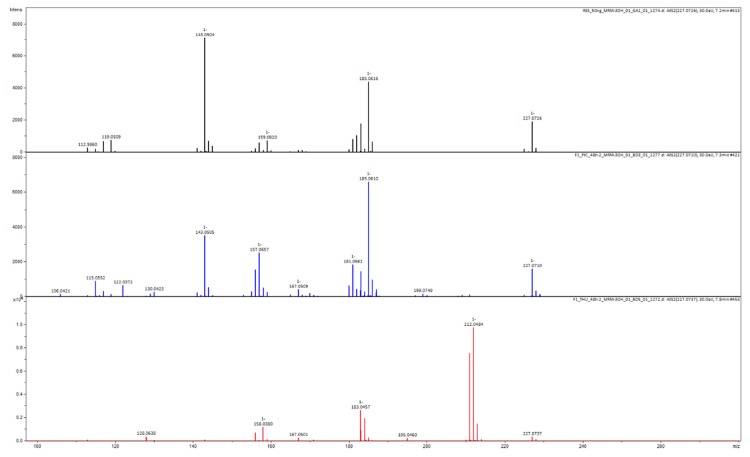
MS/MS spectra of resveratrol, 3,3′,4′-trihydroxystilbene/3′,4′,5-trihydroxystilbene and isoresveratrol.

The only metabolite of resveratrol detected in our model was dihydroresveratrol, which was not further metabolized and remained stable in the colon environment. Amounts of resveratrol and dihydroresveratrol were monitored: after 48 h, the concentration of resveratrol decreased to 3.2 ± 0.9 µg/mL (mean ± SD) from the initial 9.1 ± 4.4 µg/mL. The concentration of dihydroresveratrol rose gradually to a final concentration of 0.7 ± 0.4 µg/mL after 48 h of fermentation. The metabolism of oxyresveratrol was similar to resveratrol, and one metabolite, 2′,3,4′,5-tetrahydroxybibenzyl, formed by double bond reduction, was detected. It reached its maximum level after 24 h and then was further degraded to still unknown products. The metabolism of piceatannol was more complex. The main metabolic pathway was the double bond reduction of the connective chain, forming dihydropiceatannol, which reached its maximum level after 4 h and then was also further degraded to still unknown products. In some donors, piceatannol was dehydroxylated at one of the meta positions on ring A, forming trihydroxystilbene different than resveratrol or isoresveratrol (see Appendix A, Figure A1), and was further metabolized to 3,3′,4′-trihydroxybibenzyl. This reaction was much slower than the formation of dihydropiceatannol and occurred between 4 to 8 h of fermentation. Even though thunalbene was not observed in the samples, its demethylated metabolite, isoresveratrol (3,3’,5-trihydroxystilbene), was detected. This metabolite was stable and was not further metabolized or degraded. Batatasin III and pinostilbene did not form any metabolites and were found to be stable in the colon environment.

**Figure A2:**
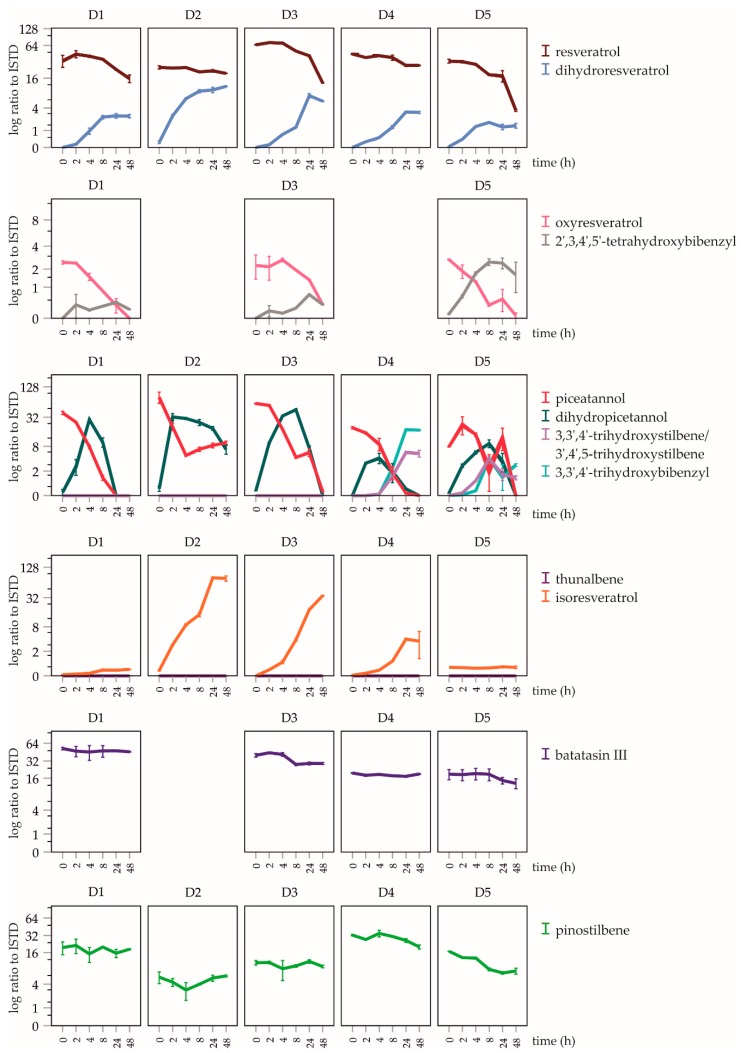
Comparison of metabolism between donors. Values are expressed as Means ± 1 SE, n = 3, technical replicates.

Strong inter-individual differences were observed among the donors, as seen in Appendix B Figure A2. Resveratrol was gradually metabolized in all samples to dihydroresveratrol, but the final concentrations varied among the donors from 77 ± 1% (mean ± SD; D2) to 11 ± 1% (D5). Oxyresveratrol was metabolized in each sample at different intensities, 100 ± 0% (D1), 84 ± 1% (D3), and 98 ± 2% (D5). Piceatannol, after 48 h of fermentation, was more than 99.6% metabolized in four of the samples, except for sample D2, where 12 ± 1% of piceatannol was still present at the end of the fermentation. Piceatannol was metabolized to dihydropiceatannol in all faecal samples, but in two out of the five cases (D4 and D5) it was metabolized to 3,3′,4′-trihydroxystilbene or 3′,4′,5-trihydroxystilbene and further to 3,3′,4′-trihydroxybibenzyl. Similarly, thunalbene was metabolized to isoresveratrol in only three out of the five faecal samples (D2, 3 and 4), while in the others no metabolites were detected.

## 3. Discussion

The current study provides new information about the biotransformation of six stilbenoids by human gut microbiota, depending on their structural molecular properties. The obtained data show that stilbenoids differ in their stability in a colonic environment. Whereas batatasin III and pinostilbene did not produce any metabolites, resveratrol, oxyresveratrol, piceatannol, and thunalbene were intensively metabolized by colon microbiota. Three main reactions were found to be ongoing in our human colon model: double bond reduction, dihydroxylation, and demethylation. An important factor in the course of the reactions was the location of the hydroxyl and methyl groups. Differences in the intensity, rate, and spectrum of metabolites were also observed among the faecal samples obtained from different donors.

The only resveratrol metabolite detected in this study was dihydroresveratrol, which is in agreement with other in vitro and in vivo studies [23,24,25]. Another study, using a similar model, described two more metabolites, 3,4′-dihydroxy-*trans*-stilbene and 3,4′-dihydroxybibenzyl (lunularin), which were detected in six out of seven faecal samples [20]. Their absence in our study might be caused by a different composition of bacterial species in faecal samples or different initial concentrations of stilbenoids, which might saturate some enzymatic catabolic pathways and change the method of metabolite formation. It is evident that the course of the catabolic reaction was affected by the inter-individual differences in the bacterial composition of the faecal samples. In our study, resveratrol had been gradually catabolized, and its final concentration, after 48 h of fermentation, ranged from 77 ± 1% (D2) to 11 ± 1% (D5). This gradual decrease contrasts with another study [20] that reported a complete degradation of resveratrol in a time frame of 2 to 24 h. This might be partly caused by different initial concentrations of resveratrol (80 µM vs. 44 µM in our study), lower inoculum, medium composition, or simply differences in the donor’s microbial composition. Previously, we reported bacterial composition in a subset of samples reported here (time points 0 and 24 h; and donors D1-D4), showing major differences between this study [26] and the study from [20]. While Faecalibacterium (12–21% of DNA) and Bacteroides (9–16% of DNA) were the most abundant group in [20], our fermentations were dominated by Clostridia at time points 0 h and 24 h [26]. Mean *Faecalibacteium prausnitzii* abundance at time point 0 h was only 2.01 ± 1.01% and Bacteroides were only 0.06 ± 0.06%. In a previous study, 43 bacteria, mostly gut-associated strains, were screened for their capacity to catabolize resveratrol [23], from which 11 strains were capable of metabolizing resveratrol by more than 20%, among them *Escherichia coli* ATCC 25922, *Bacillus cereus* NCTR-466, and *Achromobacter denitrificans* NCTR-774 had transformed resveratrol almost completely within 24 h.

Similar to resveratrol and oxyresveratrol, piceatannol was metabolized to dihydropiceatannol by colon microbiota via hydrogenation of the ethylene bridge. However, another pathway has also been detected. Faecal bacteria from some donors were able to cleave the hydroxyl group on ring A in one of the meta positions and form trihydroxystilbene, different from resveratrol or isoresveratrol, which was further dehydroxylated to 3,3′,4′-trihydroxybibenzyl.

In the present study, three derivatives of stilbenoids formed by double bond reduction were detected. Their further fate in the colon model was dependent on the position of the hydroxyl group. Dihydroresveratrol, with only one hydroxyl group on ring B in para position, was not further metabolized or degraded, and it was the end-product of resveratrol colon metabolism. The catabolite of oxyresveratrol, 2,3,3′,5′-tetrahydroxystilbene, has two hydroxyl groups in para and ortho positions on ring B. Concentration change of this metabolite was more dynamic in this model, reaching its maximum concentration at 24 h and then further degrading to still unknown products. The least stable of these metabolites was dihydropiceatannol, which had two hydroxyl groups in para and meta position on ring B. It has been shown that dihydroresveratrol could be produced as an end-product or transient intermediate, depending on the donor [20], so the high persistence of dihydroresveratrol in the present study might only be an observed effect particular to our donors. The marked influence of colon microbiota composition on the metabolism of polyphenols has also been well reported by other authors [19].

Thunalbene was not observed as a parent compound in the samples, possibly because of binding to the matrix or polymerization. However, its demethylated catabolite, isoresveratrol, was detected in three out of the five donors (D2, 3, and 4). Interestingly, batatasin III and pinostilbene, other methylated stilbenes, were demonstrably stable in the colon model. This matches results obtained in a study of pterostilbene (3,5-dimethoxy-4′-hydroxystilbene) metabolism in the colons of mice, where pinostilbene was found as its main product; however, no metabolites of pterostilbene with a reduced double bond were identified [18,27]. This result indicates that the position of the methoxy group could play an important role in its demethylation, as well as in the reduction of the ethylene bridge by intestinal microbiota.

In our previous study [26] the microbiota composition of donors D1-4 had been observed. Compared to the others, donors D1 and D2 seemed to be very atypical, with a higher representation of class Bacilli (*Streptococcaceae* family) in donor D1 and a higher representation of the *Enterobacteriaceae* family in donor D2. Due to the lack of the compounds, the full set of all six stilbenoids was fermented only by samples from donors D1, D3, and D5. Microbiota from the faecal sample of donor D1 was able to metabolize about half of the resveratrol and completely metabolize oxyresveratrol and piceatannol within 48 h, but it was not metabolizing thunalbene. Microbiota from donor D3 were effectively metabolizing resveratrol (20 ± 1% occurred after 48 h), and demethylating thunalbene to isoresveratrol, but was the least effective in metabolizing oxyresveratrol (84 ± 1% occurred after 48 h). Microbiota from donor D5 were the most effective in metabolizing resveratrol (11 ± 1% occurred after 48 h), and were able to dehydroxylate piceatannol. However, similar to donor D1, these microbiota were not able to metabolize thunalbene to isoresveratrol. Microbiota from donor D2 were the least effective in metabolizing resveratrol (77 ± 1% occurred after 48 h), and piceatannol (12 ± 1% occurred after 48 h) but seem to be the most effective in metabolizing thunalbene. Microbiota of donor D4 were not very effective in metabolizing either resveratrol or thunalbene but were as efficient as an inoculum of donor D5 by dehydroxylating piceatannol.

In conclusion, it has been shown that some stilbenoids are intensively catabolized by the colon microbiota, whereas others seem to be stable in the colon environment. In our model, the degree of substitution played an important role on the level of molecular fragility. Derivatives with the hydroxy group in the para position were less fragile than the others, and further study of their behavior in the colon is needed. The rate, intensity, and the pathways of metabolism are closely associated with colon microbiota composition. However, the role of particular bacterial species on the metabolism of stilbenoids is not clear, and thus future research should also be focused on inter-individual differences and work with a larger number of donors. To our knowledge, this is the first study investigating the metabolic fate of stilbenoids other than resveratrol in the faecal human colon model. This study has also implication on future screening assays for biological activities, so that relevant metabolites can be included.

## 4. Materials and Methods

### 4.1. In vitro Faecal Fermentation System

A slightly modified fermentation model, previously described by other authors [28,29], was used to mimic the conditions in the human colon.

#### 4.1.1. Fermentation Medium

The fermentation medium was prepared as a solution of 225 mL distilled water, 1.12 g tryptone, 56.25 μL of micromineral solution (2.64 g CaCl_2_·2H_2_O, 2 g MnCl_2_·4H_2_O, 0.20 g CoCl_2_·6H_2_O, 1.60 g FeCl_3_·6H_2_O, and distilled water up to 20 mL), 112.5 mL of macromineral solution (7.14 g of Na_2_HPO_4_·2H_2_O, 6.20 g KH_2_PO_4_, 0.60 g MgSO_4_·7H_2_O, and distilled water up to in 1 L), 112.5 mL of CO_3_ buffer (1 g NH_4_HCO_3_, 8.75 g NaHCO_3_, and distilled water up to 250 mL), and 562.5 μL of 0.1% resazurin solution. All chemicals were obtained from Merck (Darmstadt, Germany) and stored at 4 °C for up to 1 month. The prepared fermentation medium was covered with aluminum foil and stored at 4 °C until the next day.

#### 4.1.2. Sodium Phosphate Buffer and Reducing Solution

The sodium phosphate buffer for preparation of the faecal slurries was made of 1.77 g KH_2_PO_4_ in 195 mL distilled water and 3.62 g of Na_2_HPO_4_ in 305 mL distilled water (both 1/15 M). Afterward, the buffer’s pH was modified to 7.0 by hydrochloric acid and stored at 4 °C for up to one month. The reducing solution was prepared just before the experiment from 125 mg cysteine hydrochloride, 0.8 mL 1M NaOH, 125 mg Na_2_S, and distilled water up to 20 mL.

#### 4.1.3. Stilbenoid Preparation

Batatasin III, piceatannol, thunalbene, and pinostilbene were purchased from ChemFaces (China) at 98% purity; resveratrol, oxyresveratrol, and [^13^C_6_] *trans*-resveratrol were obtained from Merck (Darmstadt, Germany) at 98% purity. Stock solutions for fermentation experiments were prepared at a concentration of 10 mg/mL in DMSO (dimethylsulfoxide; Sigma-Aldrich, Prague, Czech Republic) and kept at 4 °C. Analytical standard stock solutions for LC/MS were prepared in methanol (1 mg/mL) and stored at −80 °C.

#### 4.1.4. Faecal Samples and Ethics Statement

Human faecal samples were collected in October and November 2016, at the Czech University of Life Sciences in Prague, Czech Republic from 5 healthy volunteers. These volunteers were aged 23 to 29 years, with a mean BMI of 24.6, no history of gastrointestinal disease and no antibiotic treatment for at least 3 months prior to the experiment. Female volunteers were neither pregnant nor lactating. All donors followed an omnivorous diet in their daily life and a two-day low polyphenol diet before the sample collection. All subjects gave their informed consent for inclusion before they participated in the study. The study was conducted in accordance with the Declaration of Helsinki, and the protocol was approved by the Ethics Committee of the Czech University of Life Sciences in Prague (ZEK/22/09/2017). Samples were collected into 1 L plastic container, tied in a plastic bag with GENbag anaer (Biomérieux, Lyon, France) and kept at 37 °C for 2 h maximum. Fresh faeces were homogenized in a stomacher bag for 30 s with a sodium phosphate buffer and the obtained 32% faecal slurry was filtrated through a nylon mesh.

#### 4.1.5. In Vitro Incubations

The fermentation medium and sodium phosphate buffer were boiled with cotton cups and cooled to approximately 37 °C while they were purged with nitrogen gas free of oxygen (approximately 30 min). In the end, the color of the medium changed from blue to pink. The medium’s pH was adjusted to pH 7.0 using HCl. The fermentation bottles (20 mL) were filled with 16.8 mL of medium and sealed with PTFE/aluminum caps. The reducing solution (0.8 µL) was added through the septa and, after full decolorization of resazurin, 20 µL of the tested compound and 2 mL of the faecal slurry were added. 20 µL of DMSO, instead of the tested compound, was added to the negative control vials, and 2 mL of the sodium phosphate buffer, instead of the faecal slurry, was added to the positive control vials. The incubation was carried out in a shaking water bath at 37 °C, at 100 strokes per minute. Samples (3 mL) were collected at 0, 2, 4, 8, 24, and 48 h with a syringe through the septa and stored at −80 °C until analysis.

### 4.2. LC/MS analyses

#### 4.2.1. Standards

Standards were prepared as 1% Methanol/Formic Acid solution. Stock solutions were prepared at a concentration of 10 mg/mL in DMSO (Sigma-Aldrich, Stribrna Skalice, Czech Rep) and kept at 4 °C.

#### 4.2.2. Sample Purification

A liquid-liquid extraction was used for the purification of samples. The samples from fermentations were centrifuged (5 min; 15,000 rpm/min), and 400 μL of supernatant was diluted with 2 mL of ultra-pure water (Milipore, Bedford, MA, United States of America); 20 μL of [^13^C_6_] *trans*-resveratrol solution in methanol (2 μg/mL) was added as an internal standard. Then, the samples were extracted three times by 2.5 mL ethyl acetate (VWR Chemicals, Stribrna Skalice, Czech Republic). After purification, the combined organic phase was dried under nitrogen gas and re-dissolved in 1 mL of methanol (VWR Chemicals, Stribrna Skalice, Czech Republic) with 1% formic acid (Fisher Scientific, Merelbeke, Belgium). Final samples were analyzed by LC/MS.

#### 4.2.3. LC/MS Analysis of Metabolites

Analyses were performed on a LC/MS system consisting of an UHPLC chromatograph Ultimate 3000 Thermofisher Scientific (Sunnyvale, CA, USA) coupled with quadrupole time of flight (Q-TOF) mass spectrometer with ultra-high resolution and high mass accuracy (HRAM) Impact II (Bruker Daltonics, Bremen, Germany) equipped with an electrospray ionization (ESI) source.

Chromatography was carried out on a Kinetex 1.7 µm F5 100 Å 100 × 2.1 mm column (Phenomenex, Torrance, CA, USA) using a mobile phase consisting of 0.1% formic acid (solvent A) and methanol (solvent B). The binary gradient was run as follows: 0–3 min isocratic at 20 % B, 3–6 min from 20 % to 50 % B, 6–15 min from 50% to 100 % B, and 15–20 min isocratic at 20 % B. The flow rate was kept at 0.2 mL/min, and the column oven was adjusted to 35 °C. The injected volume was 5 μL. 

The ESI source was operated in the negative mode with parameters listed in Appendix C, Table A1. The identity of each detected compound was confirmed by MS/MS fragmentation spectra collected at three collision energy levels (20, 30 and 50 eV). Data acquisition was performed using HyStar 3.2 SR4, QTOF series 4.0 (Bruker Daltonics–Germany), and Chromeleon Xpress (Thermo Fisher Scientific) software, and the obtained data were processed by DataAnalysis 4.3. and TASQ 1.4 (both Bruker Daltonics–Germany). For calibration, commercially available standards of resveratrol, dihydroresveratrol, oxyresveratrol, piceatannol, thunalbene, batatasin III and pinostilbene were used each at 6 concentration levels in the range of 20–1000 ng/mL. As an internal standard, 20 μL of *trans*-[^13^C_6_] resveratrol at a concentration of 2 μg/mL was used. 

#### 4.2.4. Statistical Evaluation

Resveratrol, thunalbene, piceatannol and pinostilbene were used in five biological repetitions. Oxyresveratrol and batatasin III were used in three and four repetitions, respectively. All samples were measured by LC/MS in triplicates. Values are expressed as a mean ± standard error. Microsoft Excel and SPSS (IBM corp.) version 25 were used for basic statistical analysis and graph creation.

## Appendix C

**Table A1:** List of the stilbenoids monitored and detected in the samples by LC/MS.

Name	Molecular Formula	Neutral Molecule Exact Mass	Measured [M − H]^−^ Exact Mass	Comparison with Standard
**monitored:**				
thunalbene	C_15_H_14_O_3_	242.0943	241.0865	YES
pinostilbene	C_15_H_14_O_3_	242.0943	241.0865	YES
piceatannol	C_14_H_12_O_4_	244.0736	243.0657	YES
oxyresveratrol	C_14_H_12_O_4_	244.0736	243.0657	YES
batatasin III	C_15_H_16_O_3_	244.1099	243.1021	YES
resveratrol	C_14_H_12_O_3_	228.0786	227.0708	YES
lunularin	C_14_H_14_O_2_	214.0994	213.0916	YES
**detected:**				
dihydroresveratrol	C_14_H_14_O_3_	230.0943	229.0865	YES
2,3′,4,5′-tetrahydroxybibenzyl	C_14_H_14_O_4_	246.0892	245.0814	NO
dihydropiceatannol	C_14_H_14_O_4_	246.0892	245.0814	NO
trihydroxystilbene	C_14_H_12_O_3_	228.0786	227.0708	NO
3,3′,4-trihydroxybibenzyl	C_14_H_14_O_3_	230.0943	229.0865	NO
isoresveratrol	C_14_H_12_O_3_	228.0786	227.0708	NO
